# Regulation of cancer cell metabolism: oncogenic MYC in the driver’s seat

**DOI:** 10.1038/s41392-020-00235-2

**Published:** 2020-07-10

**Authors:** Yang Dong, Rongfu Tu, Hudan Liu, Guoliang Qing

**Affiliations:** 1 Department of Urology, Zhongnan Hospital of Wuhan University, Wuhan, 430071 China; 2 Frontier Science Center for Immunology & Metabolism, Medical Research Institute, Wuhan University, Wuhan, 430071 China

**Keywords:** Cancer metabolism, Cancer metabolism

## Abstract

Cancer cells must rewire cellular metabolism to satisfy the demands of unbridled growth and proliferation. As such, most human cancers differ from normal counterpart tissues by a plethora of energetic and metabolic reprogramming. Transcription factors of the MYC family are deregulated in up to 70% of all human cancers through a variety of mechanisms. Oncogenic levels of MYC regulates almost every aspect of cellular metabolism, a recently revisited hallmark of cancer development. Meanwhile, unrestrained growth in response to oncogenic MYC expression creates dependency on MYC-driven metabolic pathways, which in principle provides novel targets for development of effective cancer therapeutics. In the current review, we summarize the significant progress made toward understanding how MYC deregulation fuels metabolic rewiring in malignant transformation.

## Introduction

Deregulated metabolism is an essential feature of malignant transformation. To support their relentless cell division, most cancer cells have to evolve specific metabolic adaptations that promote their survival under conditions that kill normal counterparts; this adaptation process has been termed “metabolic reprogramming”. Multiple regulatory mechanisms, either intrinsic or extrinsic, converge to alter core cellular metabolism and provide support for the increased demands of proliferating cancer cells: rapid ATP generation to maintain energy status, increased production of anabolic intermediates for macromolecule biosynthesis, and appropriate maintenance of redox homeostasis to reduce the impact of cellular reactive oxygen species (ROS).^1,2^

The regulation and dynamics of the central metabolic pathways and energy production differ between normal and malignant cells. Fast-growing, poorly differentiated tumor cells typically exhibit increased aerobic glycolysis, even in the presence of replete oxygen, by converting a majority of glucose-derived pyruvate to lactate, a phenomenon known as the Warburg effect.^3^ Because of this, tumor cells depend on glutamine anaplerosis to replenish the tricarboxylic acid (TCA) cycle intermediates for macromolecular biosynthesis and nicotinamide adenine dinucleotide phosphate production.^4^ Although aerobic glycolysis and glutamine anaplerosis endow cancer cells with the ability to generate biosynthetic intermediates, thus enabling tumor cells to proliferate faster and outnumber their normal counterparts, these metabolic alterations cannot explain all the metabolic changes that are necessary to support the requirements of cell growth. Instead, cancer cells acquire alterations to the metabolism of all four major classes of macromolecules: carbohydrates, proteins, lipids, and nucleic acids, which act in concert to support cellular biomass synthesis, and energy storage for uncontrolled proliferation and growth, rendering adaption to a variety of stressed conditions.^5,6^ Moreover, metabolic reprogramming frequently cooperates with genomic instability, chronic inflammation, and immune escape to promote tumor progression.^7^

**Fig. 1:**
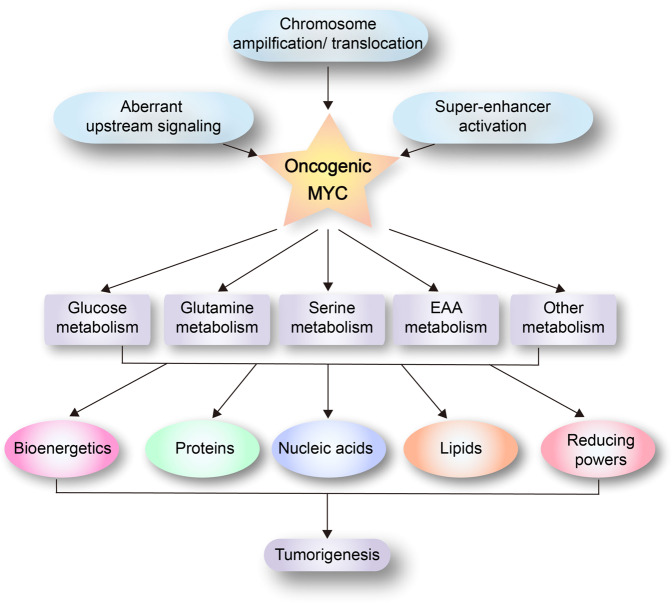
MYC coordinates global metabolic reprogramming. Deregulation of MYC frequently occurs in human cancers through a variety of mechanisms, such as chromosome amplification/translocation, super-enhancer activation, and aberrant upstream signaling. MYC deregulation rewires multiple metabolic pathways to generate energy, building blocks, and reducing power for growing tumor cells. EAA essential amino acid

Metabolic reprogramming of cancer cells is directly regulated by multiple oncogenic factors and tumor suppressors. The MYC family of oncoproteins, including MYC, MYCL, and MYCN, is an essential, master regulator of metabolic reprogramming in a broad spectrum of human cancers.^8,9^ While MYC expression is tightly regulated in normal cells, it becomes deregulated in up to 70% of all human cancers through a variety of mechanisms, such as genetic copy-number gain (chromosome amplification or translocation), super-enhancer activation, aberrant upstream signaling, and altered protein stability (Fig. 1).^10–16^ A large body of evidence demonstrates that enhanced MYC expression is a major driving force of malignant transformation, and that both MYC-driven tumors and tumors driven by other oncogenes (e.g., *K-RAS*) sustainedly depend on elevated MYC levels for growth.^17,18^ The MYC oncoproteins are “super-transcription factors” that potentially regulate the transcription of at least 15% of the entire genome.^19^ The major downstream effectors of MYC include those involved in ribosome biogenesis, mitochondrial biogenesis, protein translation, cell cycle progression, and metabolism. Accumulative evidence has shown that MYC plays an essential role in the regulation of global metabolic reprogramming, enabling rapid generation of bioenergetic substrates, and building blocks to sustain the uncontrolled cancer cell proliferation (Fig. 1).^6^

The aim of this review is to summarize the roles of MYC oncoproteins in the regulation of cancer cell metabolism, and to present the opportunities for targeting MYC-driven metabolic vulnerabilities in cancer treatment. As cancer cells acquire metabolic adaptations in response to a variety of cell-extrinsic and cell-intrinsic cues, a single model of MYC-driven tumor metabolism does not describe the sum of metabolic changes that support cell growth. Instead, MYC effects on cellular metabolism depend both on the tissue of tumor origin and on interaction with tumor microenvironment. A better understanding of this heterogeneity may enable the development and optimization of therapeutic strategies that more effectively target cancer cell metabolism.

## MYC regulation of glucose metabolism

One of the most striking characteristics of tumor metabolic reprogramming is aerobic glycolysis (the Warburg effect). Aerobic glycolysis is a typical metabolic adaption with an increasing reliance on high glucose uptake, glycolytic metabolism, and lactate production even under aerobic conditions.^3^ This biological process offers plenty of metabolic intermediates as anabolic precursors and generates energy to meet the requirement of the rapid tumor cell proliferation, especially in the hypoxic tumor microenvironment.

**Fig. 2:**
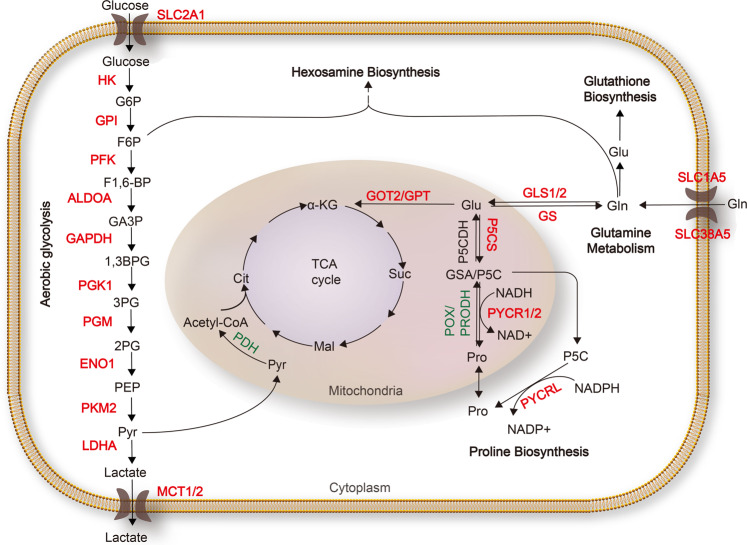
MYC regulation of glucose, glutamine, and proline metabolism. MYC promotes glucose metabolism by upregulating glucose transporters and nearly all the glycolytic enzymes. MYC ellicts glutamine addiction by promoting glutamine uptake and glutaminolysis. MYC promotes proline anabolism and represses its catabolism. Enzymes labeled in red are upregulated by MYC, and those labeled in green are suppressed by MYC. α-KG α-ketoglutarate, ALDOA aldolase A, 1,3BPG 1,3-bisphosphoglycerate, Cit citrate, ENO enolase, F1,6-BP fructose 1,6-bisphosphate, F6P fructose 6-phosphate, GA3P glyceraldehyde-3-phosphate, GAPDH glyceraldehyde-3-phosphate dehydrogenase, Gln glutamine, GLS glutaminase, Glu glutamate, GOT2 glutamate oxaloacetate transaminase, G6P glucose-6-phosphate, GPI phosphoglucose isomerase, GPT glutamine pyruvate transaminase, GS glutamine synthetase, GSA glutamic-γ-semialdehyde, HK hexokinase, LDHA lactate dehydrogenase A, Mal malate, MCT monocarboxylate transporter, P5C Δ^1^-pyyroline-5-carboxylate, P5CDH P5C dehydrogenase, P5CS P5C synthase, PDH pyruvate dehydrogenase, PEP phosphoenolpyruvate, PFK phosphofructokinase, PG phosphoglycerate, PGK phosphoglycerate kinase, PGM phosphoglucomutase, PKM2 pyruvate kinase M2, POX/PRODH proline oxidase/dehydrogenase, Pro proline, PYCR P5C reductase, Pyr pyruvate, SLC solute carrier family, Suc succinate, TCA tricarboxylic acid

MYC plays a key role in the regulation of aerobic glycolysis. MYC directly activates the transcription of almost all glycolytic genes through binding the classical E-box sequence (CACGTG; Fig. 2).^20^ Glucose transporter SLC2A1 is one of MYC targets, and upregulated by MYC to enhance glucose uptake.^21^ Chromatin immunoprecipitation assay indicated that hexokinase II (*HK2*), enolase 1 (*ENO1*) and lactate dehydrogenase A (*LDHA*) were bound by MYC on the canonical MYC-binding E-box spanning different species.^20^ MYC also modulates lactate export by inducing monocarboxylate transporters, MCT1 and MCT2, to shift toxic levels of lactate within tumor cells.^22^ Additionally, glyceraldehyde-3-phosphate dehydrogenase (*GAPDH*) and triose phosphate isomerase (*TPI*) are regulated by MYC even though no canonical E-boxes are found proximal to the promoters, suggesting that these genes are regulated by MYC indirectly.^20^

MYC is documented to activate glycolytic genes not only by transcription, but also through alternative splicing. Pyruvate kinase type M2 (PKM2) promotes the final step in aerobic glycolysis, while PKM1 seems to promote oxidative phosphorylation.^23^ Three splicing factors, heterogeneous nuclear ribonucleoprotein (hnRNP) A1, hnRNP A2, and polypyrimidine tract-binding protein are involved in *PKM* pre-mRNA alternative splicing.^23^ MYC was shown to activate transcription of these splicing factors to elevate the expression of *PKM2* over *PKM1*, hence to promote glycolysis.^23^

In addition to MYC, hypoxia-inducible factor (HIF)-1α is another critical transcription factor responsible for glycolysis in tumor cells short of oxygen supply. Both MYC and MYCN collaborate with HIF-1α to stimulate the expression of key glycolytic genes in response to hypoxia, such as *HK2* and pyruvate dehydrogenase kinase 1 (*PDK1*) in MYC-driven Burkitt’s lymphoma cells, and *LDHA* in *MYCN*-amplified neuroblastoma cells, suggesting cooperation between MYC oncoproteins and HIF-1α plays an important role in regulation of glucose metabolism in cancers.^24,25^

## MYC regulation of amino acid metabolism

While deregulated glucose metabolism is widely appreciated in many cancer types, an elevated demand for amino acids must also be met to support cell proliferation and cancer progression. Some amino acids can be synthesized by cancer cells, while other essential amino acids (EAAs) must be derived from the extracellular milieu. Moreover, many cancers also rely on access to non-essential amino acids (non-EAAs) from their environment. MYC plays an essential role in regulation of aberrant amino acid metabolism.^26,27^

### Essential amino acids

Mammalian cells, whether they are cancerous or not, have to obtain EAAs from the extracellular milieu because they are unable to produce EAAs de novo. There are a total of nine amino acids (histidine, isoleucine, leucine, lysine, methionine, phenylalanine, threonine, tryptophan, and valine) that are essential in humans. Except for histidine, lysine, methionine, and threonine, the remaining five are EAAs with large branched or aromatic side chains, collectively called large neutral EAAs (LNEAAs). EAAs not only provide fundamental building blocks for macromolecular biosynthesis, but also serve as signaling molecules to induce mammalian target of rapamycin (mTOR) activation.^28,29^

**Fig. 3:**
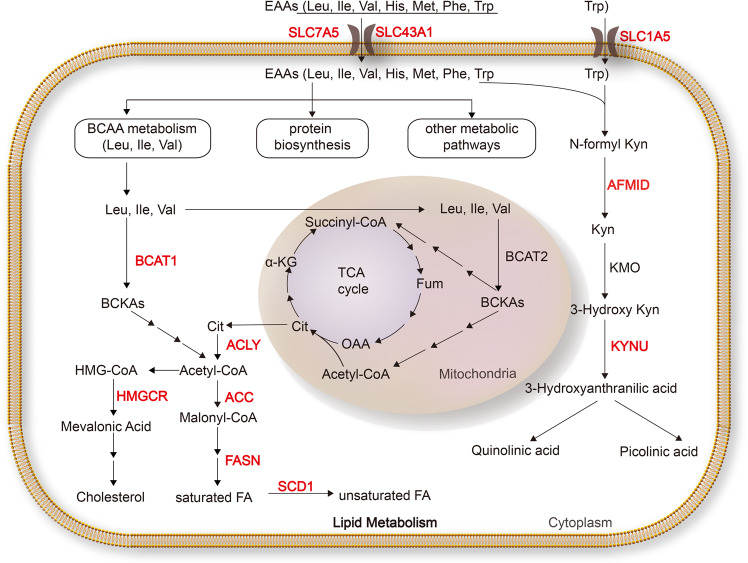
MYC regulation of essential amino acid and lipid metabolism. MYC activates critical transporters, SLC7A5, SLC43A1, and SLC1A5, to promote essential amino acid transport. BCAT1, which catalyzes the decomposition of branched amino acids, is a downstream target of MYC. MYC promotes tryptophan uptake and metabolism by the kynurenine pathway. MYC coordinates glucose, glutamine, and essential amino acid metabolism to promote fatty acid biosynthesis. Enzymes labeled in red are upregulated by MYC. ACC acetyl-coA carboxylase, ACLY ATP citrate lyase, AFMID arylformamidase, BCAA branched-chain amino acid BCAT branched-chain aminotransferase, BCKA branched-chain α-keto acid, FA fatty acid, FASN fatty acid synthase, Fum fumarate, His histidine, HMG-CoA 3-hydroxy-3-methylglutaryl-CoA, HMGCR 3-hydroxy-3-methyl-glutaryl-coenzyme A reductase, Ile isoleucine, KMO kynurenine-3-monooxygenase, Kyn kynurenine, KYNU kynureninase, Leu leucine, Met methionine, OAA oxaloacetate, Phe phenylalanine, SCD stearoyl-CoA desaturase, Thr threonine, Trp tryptophan, Val valine

As EAAs must be uptaken from external sources, transporters responsible for EAA absorbance are required to be upregulated to satisfy the requirements of cancer cells. Members of the SLC7 family (SLC7A5 and SLC7A8), the SLC43 family (SLC43A1 and SLC43A2), the SLC6 family (SLC6A14), and the SLC38 family (SLC38A1–SLC38A11) primarily mediate the uptake of EAAs.^29^ We recently identified a *MYC*-*SLC7A5/SLC43A1* signaling circuit that underlies LNEAA metabolism, MYC deregulation, mTOR complex 1 (mTORC1) activation, and tumor progression. Notably, SLC7A5/SLC43A1-mediated EAA uptake in turn stimulates MYC protein synthesis and downstream target gene transcription, leading to reprogramming of the entire metabolic processes, including glycolysis, glutaminolysis, and lipogenesis (Fig. 3).^29^

Branched-chain amino acids (BCAAs), which include leucine, isoleucine, and valine, are one class of amino acids whose metabolism has been associated with specific cancer phenotypes. BCAA metabolism can both influence multiple cancer phenotypes and serve as a marker of disease pathology.^30^ As such, BCAA metabolism and BCAA metabolic enzymes, such as the cytosolic branched-chain aminotransferase 1 (BCAT1), play key roles in the progression of different cancer types.^31^ MYC directly targets BCAT1 to upregulate its expression, and promotes BCAA catabolism involved lipid synthesis.^31–33^

Cancer cells have also a multifaceted relationship with altered tryptophan metabolism. In colonic cells, MYC promotes the expression of the tryptophan transporters (SLC7A5 and SLC1A5) and enzyme arylformamidase in the kynurenine pathway, thereby driving the conversion of tryptophan into kynurenine.^34^ Of note, high levels of kynurenine can increase the proliferation and migratory capacity of cancer cells, and help tumors escape immune surveillance.^35^

In addition to MYC, HIF-2α, the Hippo pathway effectors, the hormone receptors, and the stress response factor ATF4 were shown to upregulate SLC7A5 and/or SLC43A1 expression in multiple cancer types, including clear cell renal carcinoma, hepatocellular carcinoma, breast and prostate cancers,^36–40^ which leads to elevated EAA uptake and aggressive tumor progression. Most likely, these factors cooperate with MYC to maximize SLC7A5/SLC43A1 (and additional transporters) expression and EAA uptake in human cancers.^29^

### Glutamine

In addition to glucose, glutamine is another major nutrient for cancer cells. Glutamine provides nitrogen and carbon sources for nucleotide, amino acid, and lipid biosynthesis. Meanwhile, glutamine generates energetic products through TCA cycle anaplerosis, and maintains redox homeostasis in numerous metabolic processes.

MYC plays an important role in glutamine catabolism (Fig. 2). It promotes glutamine uptake by activation of glutamine transporters *SLC1A5* and *SLC38A5*.^41^ In human P-493 B lymphoma cells and PC3 prostate cancer cells, MYC transcriptionally inhibits miR-23a/b to enhance the translation of *GLS1* (encodes glutaminase 1, also called GLS), leading to elevated glutaminolysis.^26^ Moreover, MYCN promotes glutaminolysis via selective activation of *GLS2* (encodes glutaminase 2), but not *GLS1*, transcription in *MYCN*-amplified neuroblastomas.^42,43^ Most likely, MYC regulation of glutaminolysis strictly depends on tumor context. Differential metabolic requirements within specific cancer types might dictate the final outcome of MYC regulation of glutamine catabolism.

Apart from catabolism, MYC also paradoxically regulates glutamine anabolism (Fig. 2). Glutamine synthetase (GS, also termed glutamate-ammonia ligase) catalyzes the de novo synthesis of glutamine from glutamate and ammonia, the exact reverse reaction catalyzed by glutaminase. Interestingly, in multiple human and mouse tumor cells and cancers, MYC induces active demethylation of the *GS* promoter and its increased expression through transcriptional upregulation of thymine DNA glycosylase, promoting glutamine synthesis and glutamine-dependent nucleotide biosynthesis, amino acid transport, and cell proliferation.^44^

These results reinforce the notion that a unified model of MYC-mediated glutamine metabolism might not exist. Instead, the diversities within metabolic programs of specific cancer types can dictate by what means the proliferative rewiring is fueled, which in turn imparts heterogeneities of glutamine metabolic dependencies. This notion is further supported by previous studies that in MYC-driven liver tumors GLS1 induction was accompanied by low level of GS, while in contrast, GS was elevated in MYC-driven lung tumors.^45^ Even in a single tumor cell, MYC may simultaneously promote both GS and GLS expression, as these two reactions occur at different subcellular compartments: glutaminolysis predominantly in mitochondria, whereas glutamine synthesis in the cytosol.

### Proline

Proline is the only proteinogenic secondary amino acid with its α-amino group within a pyrrolidine ring. Proline biosynthesis is increased in multiple cancer cell lines, where it is proposed to coordinate metabolic reprogramming of glucose, glutamine, and pyridine nucleotides.^46^ As such, proline starvation or inhibition of proline biosynthetic enzymes impaired clonogenic and tumorigenic potential of a subset of cancer cells.^47^

Comprehensive clinical data indicate that oncogenic MYC is correlated with proline metabolism and tumorigenic potential in a subset of cancer cells, such as the aggressive subclass of luminal breast cancer.^5^ Also, some MYC-induced neoplastic phenotypes are attributed to MYC regulation of proline metabolism.^48^

MYC promotes proline biosynthesis from glutamine by upregulating critical proline synthetic enzymes at both protein and mRNA levels, such as P5C synthase (P5CS) and P5C reductase (PYCR; Fig. 2).^46,48^ In some proline-dependent tumor cells, oncogenic MYC activated P5CS and PYCR expression to enhance glutamine-to-proline biosynthesis, thus alleviating ER stress and promoting cellular homeostasis in proline-deprived conditions.^47^ Meanwhile, MYC indirectly suppresses the expression of proline oxidase/proline dehydrogenase (POX/PRODH), which catalyzes the first step in proline catabolism. In P-493 Burkitt’s lymphoma cells, MYC transcriptionally upregulates miR-23b* to decrease the translation of *POX/PRODH*, leading to the inhibition of proline catabolism.^46,48^

In conclusion, MYC facilitates proline synthesis by regulating a series of metabolic enzymes in both anabolism and catabolism, and promotes interchange of metabolic intermediates from interconnected pathways in part through proline metabolism (Fig. 2).

### Serine and glycine

Serine and glycine are two non-EAAs that provide biomass synthesis precursors and maintain redox homeostasis, as well as refueling one-carbon metabolism. Serine also functions as an allosteric activator of PKM2, which enhances PKM2 enzymatic activity to promote glycolysis.^49^ As such, serine and glycine metabolism is often aberrant in cancers.

**Fig. 4:**
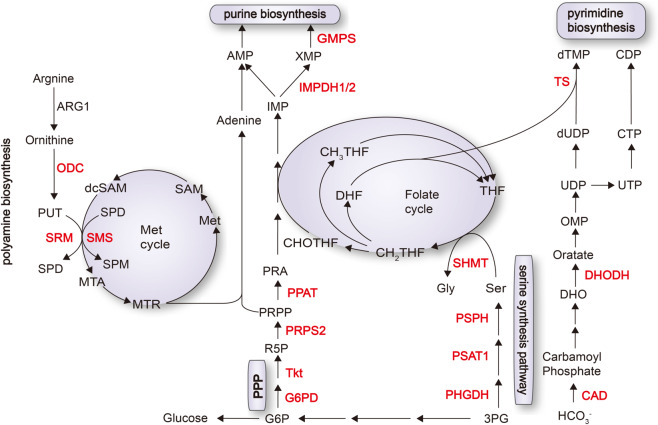
MYC coordinates multiple metabolic events to activate nucleotide metabolism. MYC activates ODC, SMS, and SRM to promote polyamine biosynthesis. MYC drives nucleotide biosynthesis (1) by facilitating generation of PRPP, which offers skeleton for de novo purine and pyrimidine biosynthesis; (2) by facilitating generation of serine and glycine, which offers one-carbon unit for folate cycle; and (3) by directly inducing enzymes involved in de novo nucleotide biosynthesis. Enzymes labeled in red are upregulated by MYC. ARG1 arginase 1, AMP adenosine monophosphate, CAD carbamoyl-phosphate synthetase; CDP cytosine diphosphate; CHOTHF formyl tetrahydrofolate; CH2THF 5,10-methylene tetrahydrofolate; CH3THF methyl tetrahydrofolate, CTP cytosine triphosphate, dcSAM decarboxylated S-adenosyl-methionine, DHO dihydroorotate, DHODH dihydroorotate dehydrogenase, DHF dihydrofolate, dUDP deoxyuridine diphosphate, dTMP deoxythymidine monophosphate, Gly glycine, GMPS guanosine monophosphate synthetase, G6PD glucose-6-phosphate dehydrogenase, IMP inosine monophosphate, IMPDH inosine-5′-monophosphate dehydrogenase, MTA 5′-methylthioadenosine, MTR 5-methylthioribose-1-phosphate, ODC ornithine decarboxylase, OMP orotate monophosphate, PHGDH phosphoglycerate dehydrogenase, PPAT phosphoribosyl pyrophosphate amidotransferase, PPP pentose phosphate pathway, PRA 5-phosphoribosylamine, PRPP phosphoribosyl pyrophosphate, PRPS pyrophosphate synthetase, PSAT phosphoserine aminotransferase, PSPH phosphate ester hydrolysis, PUT putrescine, R5P ribose 5-phosphate, SAM S-adenosyl methionine, Ser serine, SHMT serine hydroxymethyltransferase, SMS spermine synthase, SPD spermidine, SPM spermine, SRM spermidine synthase, THF tetrahydrofolate, Tkt transketolase, TS thymidylate synthase, UDP uridine diphosphate, UTP uridine triphosphate

MYC is well known to enhance the serine biosynthesis pathway by transcriptional activation of almost all the involving enzymes, such as 3-phosphoglycerate dehydrogenase (PHGDH), phosphoserine aminotransferase (PSAT1), and phosphoserine phosphatase (PSPH), and finally increases the glutathione (GSH) production and nucleotide synthesis to promote tumorigenesis (Fig. 4).^27^

Besides anabolism, MYC also upregulates serine hydroxymethyltransferase 2 (SHMT2) to promote serine catabolism and generate glycine and one-carbon unit (Fig. 4).^50^ In this regard, a previous study showed SHMT2 partially rescued the growth defects of MYC-null fibroblast cells.^51^ Paradoxically, SHMT2 is dispensable in MYC-driven lymphomagenesis and colorectal adenomagenesis.^52^ Thus, the functional link between MYC and SHMT2 may be cell context dependent.^52^ MYC was also shown to cooperate with HIF-1α or ATF4 to activate PHGDH, PSAT1, PSPH, and SHMT2 expression and serine/glycine biosynthesis, favoring tumor cell growth under stressed conditions.^53^

## MYC regulation of lipid metabolism

Elevated lipid synthesis is required for cell membrane biogenesis in rapidly proliferating tumor cells. In addition, fatty acids are very important for energy storage and production of signaling molecules. MYC has emerged as a key player in stimulating both fatty acid/cholesterol synthesis and fatty acid oxidation (FAO).^33^

MYC was first discovered to enhance fatty acid synthesis in rat fibroblasts, and multiple follow-up studies confirmed this regulation in prostate cancers, MYC-driven lymphoma tumors, MYC-driven hepatocellular carcinomas, and renal cell carcinomas.^5,54–57^ MYC promotes citrate production, which is the precursor of de novo fatty acid synthesis, through upregulation of a series of genes involved in TCA cycle driven from glucose and glutamine metabolism. Besides, MYC also activates the expression of ATP citrate lyase, acetyl-CoA carboxylase (ACC/ACACA), fatty acid synthase (FASN), and stearoyl-CoA desaturase (SCD), which are related to fatty acid synthesis (Fig. 3).^33,58^

MYC also regulates fatty acid synthesis through interactions with discrete master regulators. MondoA is a nutrient-sensing transcription factor.^59^ In multiple MYC-driven tumors, MYC appears to facilitate fatty acid biosynthesis through MondoA and ultimately activates the key enzymes SCD and FASN.^59^ MYC also induces the sterol-response element-binding protein 1 (SREBP1) expression, and acts in conjunction with SREBP1 to synergistically activate the transcription of fatty acid synthesis genes.^58^

In addition to fatty acids, MYC effectively reprograms cholesterol metabolism by upregulation of 3-hydroxy-3-methyl-glutaryl-coenzyme A reductase (HMGCR), the rate-limiting enzyme of cholesterol synthesis during malignant transformation.^60^ Moreover, it appeared that HMGCR is necessary for MYC phosphorylation and activation in some MYC-driven tumor models,^61^ arguing that a feedforward activation circuit between MYC and HMGCR promotes metabolic reprogramming and tumorigenesis.

Paradoxically, in certain tumor context, MYC also promotes FAO, which belongs to one of the mitochondrial metabolic pathways for energy production in eukaryotic cells.^33,62^ In *MYCN*-amplified neuroblastoma, MYCN inhibition appeared to decrease the expression of several essential enzymes involved in FAO, such as ETFA (ethyl trifluoroacetate), HADHA (hydroxyacyl-CoA dehydrogenase trifunctional multienzyme complex subunit alpha), and HADHB (hydroxyacyl-CoA dehydrogenase trifunctional multienzyme comple subunit beta).^62^ MYCN inhibition also leads to a disordered structure and reduction in components of mitochondrial respiratory chain, indirectly interfering FAO efficiency.^62^ Similar to neuroblastoma, in FL5.12 pre-B cells and MYC-driven triple-negative breast cancer cells, MYC also promotes FAO.^63–65^ In human mammary epithelial cells that express oncogenic levels of MYC, MYC promotes both CD36 expression at the plasma membrane and CPT1A/CPT2 expression at inner mitochondrial membrane to take up fatty acids that are destined for oxidation in the mitochondria.^65^ Moreover, MYC alters calcium (Ca^2+^) signaling and then promotes FAO by activating a Ca^2+^ -CAMKK2-AMP-activated kinase (AMPK) axis.^65^ In contrast, in rat fibroblasts, MYC suppresses FAO by downregulating the similar series of critical enzymes, such as HADHA, HADHB, ACADL (acyl-CoA dehydrogenase, long-chain), and ACADVL (acyl-CoA dehydrogenase, very long-chain).^33^ Most likely, whether MYC promotes or suppresses FAO is a cell context-dependent event.

## MYC regulation of nucleotide metabolism

Nucleotide is the basic constituent unit of ribonucleic acid and deoxyribonucleic acid, and is the precursor of nucleic acid synthesis in vivo. It participates in the basic life activities of organism, such as heredity, development, and growth. Nucleotide is essential to maintain the uncontrolled proliferation and metabolic reprogramming of cancer cells.^6,66,67^

MYC enhances nucleotide synthesis by inducing a series of genes involved in this process, further to prepare the cells ready for cell cycle transition. Meanwhile, MYC upregulates glucose-6-phosphate dehydrogenase and transketolase in pentose phosphate pathway (PPP) to generate ribose 5-phosphate.^68^ MYC also induces PRPS2 (phosphoribosyl pyrophosphate synthetase 2) to generate phosphoribosyl pyrophosphate, which offers skeleton for de novo purine synthesis, as well as pyrimidine salvage pathways.^66,69^

In purine synthesis, the purine ring formation also needs aspartate, glycine, and glutamine as the carbon and nitrogen donors. MYC facilitates nitrogen introduction through directly activation of the catalytic enzymes phosphoribosyl pyrophosphate amidotransferase (PPAT) and phosphoribosyl aminoimidazole succinocarboxamide synthetase.^67,70,71^ MYC also facilitates additional targets, such as inosine monophosphate dehydrogenase 1 and 2, involved in purine biosynthesis.^66^ In pyrimidine synthesis, MYC transcriptionally activates the carbamoyl-phosphate synthetase (*CAD*) gene, which encodes an enzyme catalyzing the first three steps in pyrimidine biosynthesis. Meanwhile, MYC coordinately upregulates dihydroorotate dehydrogenase and thymidylate synthase (TS) to increase the dNTP pools within tumor cells.^6,66^

Besides direct regulation of nucleotide synthetic genes, MYC alternatively enhances one-carbon metabolism and folate cycle, which participate in de novo nucleotide synthesis.^57^ MYC upregulates SHMT to promote one-carbon unit into tetrahydrofolate (THF), in order to participate in folate cycle.^51,57^ Metabolites of folate cycle are required for both purine and pyrimidine synthesis. For instance, N10-formyl-THF, contributes to purine ring formation, and N5,N10-methylene-THF is the methyl donor for dTMP generation, with catalyzing by the MYC-driven TS.^72^ Moreover, a critical intermediate of MYC-induced polyamine metabolism, 5′-methylthioadenosine, can also be utilized for pyrimidine salvage pathways.^73^

Collectively, MYC acts in concert with a series of metabolic pathways, such as PPP, one-carbon metabolism, folate cycle, and polyamine metabolism, to promote nucleotide synthesis (Fig. 4).

## MYC regulation of polyamine biosynthesis

Polyamines, including putrescine, spermidine, and spermine, are present in all living mammalian cells and mainly come from synthesis in vivo and food that contains high polyamine content. They are essencial for normal cell growth, participating in many fundamental processes of cell growth and survival, such as protein and nuleic acid synthesis, chromatin structure stabilization, and oxidative damage protection. Polyamine metabolism is frequently deregulated in cancer and elevated polyamine levels are necessary for transformation and tumor progression.

MYC promotes polyamine biosynthesis by upregulating multiple enzymes, such as ornithine decarboxylase (ODC) that catalyzes ornithine into putrescine. MYC directly improves the expression and activity of ODC to increase putrescine, which is also the precursor of spermidine and spermine synthesis.^74^ Moreover, MYC stimulates spermine synthase and spermidine synthase to promote spermidine and spermine biosynthesis (Fig. 4).^75^ This MYC-mediated regulation of polyamine biosynthesis has been confirmed in multiple cancers, such as leukemias, lung carcinomas, neuroblastomas, and breast cancers.^76–79^ Recent studies revealed that mTORC1 stabilizes pro-S-adenosyl methionine (AdoMet) decarboxylase (pro-AdoMetDC), leading to increased AdoMetDC to further promote polyamine biosynthesis in prostate cancer. MYC has been linked to mTORC1 activation by increasing nutrient import.^80,81^ As such, MYC may indirectly impact polyamine biosynthesis through mTOR pathway.

## Metabolic regulation of MYC expression and transcriptional activity

**Fig. 5:**
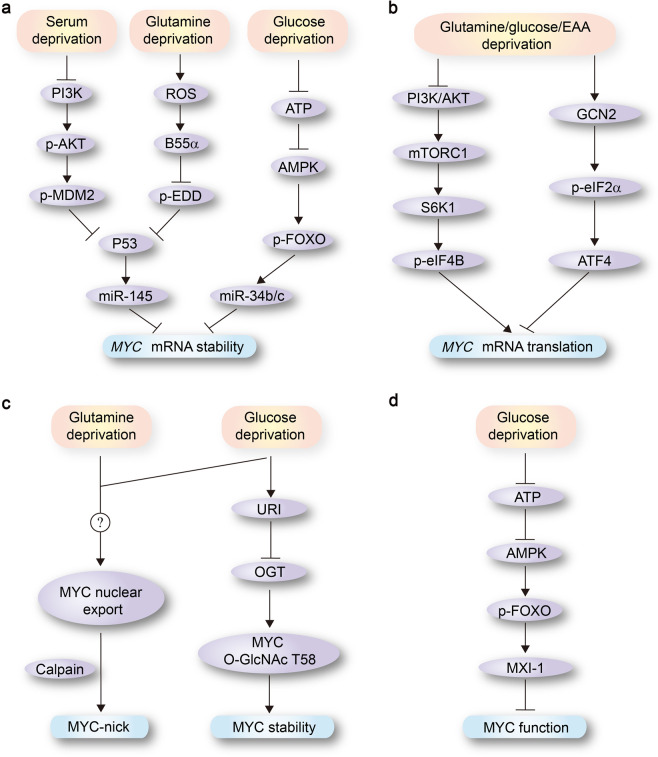
Metabolic regulation of MYC expression and transcriptional activity. Expression of MYC is downstream of multiple control mechanisms, as shown in **a**–**d**, which are regulated by nutrient levels and respond to metabolic stress. See text for more details. AMPK AMP-activated protein kinase, B55α also termed Ppp2r2a, a specific B subunit of PP2A, EDD E3 identified by differential display, eIF eukaryotic initiation factor, FOXOs forkhead transcription factors of the O class, GCN2 general control non-derepressible-2, OGT O-linked N-acetylglucosamine transferase, PI3K phosphatidylinositol 3-kinase, S6K1 effector S6 kinase 1, URI unconventional prefoldin RPB5 interactor

As discussed above, deregulated expression of MYC promotes proliferation and growth of cancer cells, and alters intermediary metabolism to match the enhanced demand for anabolic metabolites. Conversely, cancer cells need to modulate MYC expression and/or function according to the availability of nutrients, in order to avoid a metabolic collapse for better survival under poor nutrient conditions (e.g., glucose, glutamine, or EAA shortage). Thus, expression of MYC is downstream of multiple control mechanisms that are regulated by nutrient levels and respond to metabolic stress (Fig. 5).

### Metabolic controls of *MYC* mRNA stability

The tumor suppressor p53 plays an important role in sensing regulation of *MYC* mRNA stability by nutrients. Deprivation of serum and glutamine leads to p53 activation respectively through modulation of the PI3K/AKT/MDM2 pathway and the ROS/B55α/EDD pathway.^82,83^ Activated p53 directly induces the expression of miR-145, which specifically targets the 3′-UTR of *MYC* and induces *MYC* mRNA degradation (Fig. 5a).^83^

A second example of the interplay between *MYC* mRNA stabilization and metabolism is provided by the forkhead box O (FoxO) family of transcription factors. FoxO transcription factors are activated in response to a variety of metabolic stress.^84^ Following increase in the AMP/ATP ratio upon nutrient deprivation activates the AMPK, and the active AMPK phosphorylates FoxO3 on multiple sites and promotes its transcriptional activity.^84^ FoxO3 activation induces *MYC* mRNA destabilization similarly through miR-145 and miR-34b/c induction (Fig. 5a).^85,86^

### Metabolic controls of *MYC* mRNA translation

A paradigm example that controls *MYC* mRNA translation is the mTORC1 pathway, an essential nutrient-sensing pathway in mammalian cells. The activity of mTORC1 depends on both the availability of nutrients and on the cellular energetic status. Conversely, mTORC1 activation enhances *MYC* mRNA translation by S6K1-dependent phosphorylation of eIF4B, which is critical to unwind the 5′-UTR of *MYC* mRNA (Fig. 5b).^87^

In addition to mTORC1, we recently identify that increased EAA availability enhances *MYC* mRNA translation in part through attenuation of the GCN2-eIF2α-ATF4 stress response pathway (Fig. 5b).^29^ In response to EAA shortage, GCN2 promotes eIF2α phosphorylation at serine 51. Phospho-eIF2α then binds eIF2B in a nonfunctional complex that suppresses the translation initiation of most mRNAs, especially those harboring motifs with the G-quadruplex and/or the terminal oligopyrimidine (TOP) structures in their 5′-UTRs.^88,89^ Of note, we identify that both *MYC* and *MYCN* mRNAs contain multiple G-quadruplex and TOP structures in their 5′-UTRs.

Dejure and colleagues recently show that, in colorectal cancer cells, translation of MYC is controlled by glutamine via a sequence element within the 3′-UTR of *MYC* mRNA.^90^ Surprisingly, this regulatory sequence does not respond to TCA cycle intermediates, but to intracellular levels of glutamine-derived adenosine nucleotides.^90^ The precise mechanisms whereby adenosine levels regulate *MYC* translation upon glutamine deprivation remain to be resolved.

### Metabolic controls of MYC stability

A critical step in MYC degradation involves phosphorylation of the threonine 58 (T58) residue by glycogen synthase kinase 3β (GSK3β). Of note, O-glcNAcylation of T58 prevents GSK3β-mediated T58 phosphorylation and promotes MYC stabilization.^91^ In hepatocarcinoma cells, glucose maintains a heterotrimeric URI (unconventional prefoldin RPB5 interactor)/OGT (O-linked N-acetylglucosamine transferase)/PP1γ (protein phosphatase 1γ) complex, where URI acts as a rheostat maintaining the OGT enzymatic activity (Fig. 5c).^92^ Glucose depletion induces URI phosphorylation, which inhibits OGT-mediated T58 O-glcNAcylation and promotes MYC proteasomal degradation.^92^

Interestingly, glucose deprivation not only regulates MYC levels via proteasomal degradation, but also induces calpain-mediated proteolysis of MYC, which results in the formation of a truncated protein localized in the cytosol (MYC-nick) (Fig. 5c).^93^ MYC-nick comprises the N-terminal region of MYC, but lacks the nuclear localization signal and the DNA-binding domain, thus transcriptionally inactive.^93^

MYC is an integral part of the extended network comprising MAX, MXD, and Mondo proteins. The Mondo transcription factor MondoA represents a nutrient-sensing branch of this network.^94^ Since MYC drives the uptake of glucose by competing with MondoA and inhibiting TXNIP expression, one could speculate that the increased degradation of MYC upon glucose deprivation could positively regulate MondoA-TXNIP axis, thus contributing to maintain the cancer cells in a metabolically inactive state.^95^

### Metabolic controls of MYC function

A paradigm example that controls MYC function upon metabolic stress is the FoxO family of transcription factors. FoxO proteins antagonize MYC function via several mechanisms. Non-phosphorylated FoxO proteins directly block the loading of RNA polymerase II (RNAPII) to the promoters of multiple MYC target genes, thereby blunting the ability of MYC to promote transcriptional elongation by RNAPII.^96^ FoxO3A can transactivate the MYC antagonist and transcriptional repressor MXI-1, which competes for MAX dimerization to bind and inhibit MYC target genes (Fig. 5d).^97^ FoxO3A can also inhibit mitochondrial biogenesis by directly antagonizing the MYC’s ability to activate genes involved in mitochondrial function.^98^ In principle, repression of MYC-dependent gene expression by FoxO proteins would promote metabolic adaptation of cancer cells to stressed conditions.

## Targeting MYC-driven metabolic reprogramming

The ubiquity of MYC deregulation in cancer makes it an attractive therapeutic target with broad clinical potential. Indeed, multiple mouse models have demonstrated that even transient inactivation of MYC elicits tumor regression, suggesting that regulation of oncogenic MYC could be harnessed to treat cancer patients.^17,18,99^ However, MYC lacks a specific active site for small molecules, making it difficult to functionally inhibit its activities using strategies similar to those used for kinases. In addition, as a transcription factor, MYC is localized and functions in the cell nucleus. Thus, it’s very difficult to make MYC antibodies and get them to function in the nucleus for cancer treatment. Moreover, MYC is essential for normal development. For example, both MYC and MYCN promote hematopoietic stem cell survival. In the hematopoietic compartment, MYC is required at early stages of both B cell and T cell development.^100,101^ In particular, T cell proliferation and fate determination are directed by differential levels of MYC protein.^102^ In principle, direct inhibition of MYC could cause severe “ontarget” toxicity to normal tissues.

To overcome these obstacles, alternative approaches to indirectly abrogate MYC oncogenic functions have been extensively investigated. The pleiotropic roles of MYC in regulation of cancer cell metabolism have promoted evaluation of inhibiting metabolism, as selective therapeutic opportunities. We herein summarize the existing therapeutic opportunities aimed at targeting MYC-driven metabolic pathways for cancer therapy (Table 1).

**Table 1:** Summary of representative inhibitors targeting cancer cell metabolism

Target	Compound names	Clinical testing	References
*Glycolysis*			
HK2	2-DG	Phase 1/2/3 in solid tumors	^103^
LDHA	FX11	Preclinical testing only	^104^
MCT1	AZD3965	Phase 1 in solid and hematologic tumors	^105^
*Amino acid metabolism*			
GLS	CB-839	Phase 1/2 in solid and hematologic tumors	^106^
SLC7A5	JPH203 (KYT-0353)	Preclinical testing only	^107^
*Nucleotide metabolism*			
PPAT	6-MP; 6-TG	FDA approved	
TS	5-FU	FDA approved	
RNR	Gemcitabine	FDA approved	
DHFR	Methotrexate	FDA approved	
*Lipid metabolism*			
ACC	ND-646	Preclinical testing only	^112^
FASN	TVB-2640	Phase 1/2 in solid tumors	^113^
HMGCR	Lovastatin; simvastatin; atorvastatin	Phase 1/2/3 in solid tumors; FDA approved to lower cholesterol	^114,115^
*Polyamine metabolism*			
ODC	DFMO (eflornithine)	Phase 1/2/3 in solid tumors; FDA approved for treatment of *Trypanosoma brucei gambiense* and excessive facial hair growth in women	^116^

*2-DG* 2-deoxyglucose, *DHFR* dihydrofolate reductase, *5-FU* 5-fluorouracil, *6-MP* 6-mercaptopurine, *RNR* ribonucleotide reductase, *6-TG* 6-thioguanine

### Targeting glucose metabolism

Selective targeting of tumor glucose metabolism has long been considered as an attractive therapeutic strategy. MYC invariably promotes expression of critical enzymes involved in aerobic glycolysis, such as HK2 and LDHA, making cancer cells more vulnerable to glycolysis inhibition. 2-Deoxyglucose, an analog of glucose that binds and inhibits HK2, has yielded promising antitumor activity in vitro and in vivo.^103^ Unfortunately, its efficacy in clinic is markedly attenuated by the presence of large amount of its natural counterpart, glucose, in circulation. Targeting lactate metabolism by FX11, a small-molecule inhibitor of LDHA, markedly inhibited MYC-driven lymphoma progression without prominent side effects in mice.^104^ However, homozygous LDHA mutation in germline did not inhibit either initiation or progression of MYC-induced B cell lymphoma, raising concerns on the feasibility of targeting LDHA as MYC-selective therapeutics.

Aerobic glycolysis produces excessive lactate that is toxic to tumor cells. MYC modulates lactate export by inducing MCT1/MCT2 expression to shift toxic levels of lactate within tumor cells. Therefore, a potential, effective strategy is to block MYC-driven lactate export by MCT1/MCT2 inhibitors. Of note, clinical trials of the MCT1 inhibitor AZD3965 in diffuse large B cell lymphoma and Burkitt’s lymphoma, two typical MYC-driven cancer types, are currently ongoing.^105^

### Targeting amino acid metabolism

Tumor cells have a notably increased demand for amino acids to provide substrates for biomass synthesis, energy production, and redox homeostasis. Oncogenic levels of MYC induce a transcriptional program that promotes glutaminolysis and triggers cellular addiction to glutamine as a bioenergetic substrate. As such, MYC-driven cancers frequently exhibit strict dependency on glutamine metabolism. Inhibitors of glutaminase or transaminase have shown the therapeutic efficacy in multiple MYC-driven tumor models, and a representative glutaminase inhibitor, CB-839, is currently under clinical trials for patient treatment.^106^

*MYC* and *SLC7A5* constitute a feedback loop to amplify *MYC* transcriptional program, and sustain EAA metabolism in tumor cells.^29^ In principle, therapeutic targeting of *SLC7A5* would offer an opportunity to unleash the functional association between MYC and *SLC7A5*, leading to tumor suppression. JPH203 (also known as KYT-0353), a specific *SLC7A5* inhibitor,^107^ can be evaluated as a MYC-selective cancer therapeutics in the future clinical trials.

### Targeting nucleotide metabolism

Inhibitors of nucleotide metabolism, also known as antimetabolites, are small molecules that resemble nucleotide metabolites and often inhibit the activity of enzymes involved in nucleotide biosynthesis. They have been successfully used in modern chemotherapy regimens to increase cancer patient survival and, in some cases, to help cure the disease.^108–110^

Purine analogs 6-mercaptopurine and 6-thioguanine are inhibitors targeting PPAT, which catalyzes the first step in de novo purine biosynthesis.^108^ 5-Fluorouracil, a synthetic analog of uracil, is a traditional chemotherapy drug against multiple cancers by inhibition of TS.^109^ Gemcitabine (hydrochloride), an inhibitor of ribonucleotide reductase, activates checkpoint signaling pathways and induces replication stress.^110^ Methotrexate, a broadly used antitumor drug agent, inhibits dihydrofolate reductase, which involves anti-folates.^111^ All these drug targets are activated by MYC and are critical for MYC-driven metabolic reprogramming, thus antimetabolites against them should exhibit better therapeutic efficacy in MYC-driven cancers.

### Targeting lipid metabolism

MYC is a key player in regulation of lipid metabolic reprogramming. ACC, FASN, and HMGCR, three key enzymes for lipid metabolism, are significantly activated by MYC. ND-646, an allosteric inhibitor of ACC that prevents ACC dimerization and subsequently suppresses fatty acid synthesis, has shown efficacy in mouse models of lung cancer.^112^ TVB-2640 is a highly potent, selective, and reversible first-in-class inhibitor of FASN. Its monotherapy and in combination with paclitaxel have entered the clinical trial stage.^113^ Lovastatin, simvastatin, and atorvastatin are specific HMGCR inhibitors that have been FDA approved to lower cholesterol.^114,115^ Targeting these enzymes may be a therapeutic alternative for MYC-driven cancers.

### Targeting polyamine metabolism

Polyamine metabolism is frequently deregulated in malignant transformation. The metabolic pathway of polyamines provides rational drug targets. MYC promotes polyamine biosynthesis by upregulating multiple enzymes, such as ODC that catalyzes ornithine into putrescine.^74^ 2-Difluoromethylornithine (DFMO) is one of the most widely used inhibitors of ODC.^116,117^ It competes the active site with substrate of ODC, and finally covalently bounds with ODC, leading to permanent inactivation. Multiple preclinical data support DFMO-based therapies may achieve anticancer efficacy with deregulated MYC signaling, such as refractory and high-risk neuroblastomas.^118^ Theoretically, DFMO, alone or in combination with other agents, could be effective in MYC-driven cancers.

## Conclusion and perspectives

Deregulation of the *MYC* oncogene produces MYC protein that regulates almost every aspect of cancer cell metabolism, contributing to the acquisition of building blocks essential for cancer cell growth and proliferation. Because of its potent oncogenic activity and widespread deregulation in tumors, MYC has long been made a tempting target for anticancer drug development. However, pharmacological strategies capable of directly targeting MYC remain elusive. We herein described multiple potential pharmacological approaches to indirectly hijacking MYC from metabolic perspectives (Table 1). It should be noted that the multifaceted function of MYC in regulation of cancer cell metabolism opens up exciting possibilities for combined targeting strategies that may achieve better therapeutic responses. These approaches could be translated as a strategy to move forward in future patient care, as patients with MYC deregulation are likely to respond.

The significant progress made toward understanding how particular nutrients (glucose, glutamine, etc.) fuel metabolic rewiring during tumorigenesis has rekindled immense enthusiasm in examination of inhibitors/drugs targeting metabolic adaptation, as selective cancer therapeutics. However, caution should also be taken because it remains unclear as to which aspects of cell metabolism could represent a realistic, targetable vulnerability of tumor cells in comparison with normal counterparts. It should be noted that cancer cells acquire metabolic adaptations in response to a variety of cell-extrinsic and cell-intrinsic cues, thus, MYC effects on cellular metabolism depend both on the tissue of tumor origin and on interaction with tumor microenvironment. A better understanding of these metabolic diversities will improve our ability to define their contribution to aggressive tumor progression.

In conclusion, we present mechanistic insight into MYC regulation of cancer cell metabolism, and provide potential approaches to selectively targeting MYC-overexpressing tumors that are resistant to routine chemotherapies, given the ongoing development of inhibitors against critical metabolic pathways as promising anticancer drugs.
